# Enhanced photocatalytic degradation of Rhodamine B using polyaniline-coated XTiO_3_(X = Co, Ni) nanocomposites

**DOI:** 10.1038/s41598-024-83610-1

**Published:** 2025-01-28

**Authors:** Mariyem Abouri, Abdellah Benzaouak, Mohamed Elouardi, Lahcen El Hamdaoui, Fatima Zaaboul, Khalil Azzaoui, Belkheir Hammouti, Rachid Sabbahi, Shehdeh Jodeh, Mohammed Alaoui El Belghiti, Adnane El Hamidi

**Affiliations:** 1 Laboratory of Spectroscopy, Molecular Modeling, Materials, Nanomaterial, Water and Environment Laboratory, Faculty of Sciences, Mohammed V University in Rabat, Avenue Ibn Battouta, BP1014, Agdal, Rabat, Morocco; 2 Laboratory of Materials, Nanotechnologies and Environment, Center of Sciences of Materials, Faculty of Sciences, Mohammed V University in Rabat, Avenue Ibn Battouta, BP:1014, 10000 Rabat, Morocco; 3 Laboratory of Spectroscopy, Molecular Modeling, Materials, Nanomaterials, Water and Environment, Environmental Materials Team, ENSAM, Mohammed V University, B.P. 765, Agdal, Rabat, 10090 Morocco; 4 Engineering Laboratory of Organometallic, Molecular Materials and Environment, Faculty of Sciences, Sidi Mohamed Ben Abdellah University, Fes, 30000 Morocco; 5 Euromed Research Center, Euromed Polytechnic School, Euromed University of Fes, Eco-Campus, Fes Meknes Road, UEMF, Fes, 30030 Morocco; 6 Laboratory of Industrial Engineering, Energy and the Environment (LI3E) SUPMTI, Rabat, Morocco; 7 Research Team in Science and Technology, Higher School of Technology, Ibn Zohr University, Quartier 25 Mars, P.O. Box 3007, Laayoune, 70000 Morocco; 8 Department of Chemistry, An-Najah National University, P. O. Box 7, Nablus, Palestine

**Keywords:** Polyaniline, Perovskites, Nanocomposites, Photodegradation, Rhodamine B, Chemical biology, Ecology, Environmental sciences, Chemistry, Materials science

## Abstract

In this study, novel polyaniline-coated perovskite nanocomposites (PANI@CoTiO_3_ and PANI@NiTiO_3_) were synthesized using an in situ oxidative polymerization method and evaluated for the photocatalytic degradation of Rhodamine B (RhB) a persistent organic pollutant. The nanocomposites displayed significantly enhanced photocatalytic efficiency compared to pure perovskites. The 1%wt PANI@NiTiO_3_ achieved an impressive 94% degradation of RhB under visible light after 180 min, while 1wt.% PANI@CoTiO3 reached 87% degradation under UV light in the same duration. X-ray diffraction (XRD) confirmed that the crystalline structures of CoTiO_3_ and NiTiO_3_ remained intact post-polymerization. At the same time, Fourier transform infrared spectroscopy (FTIR) verified the successful deposition of PANI through characteristic functional group vibrations. Diffuse reflectance spectroscopy (DRS) revealed reduced band gaps of 2.63 eV for 1wt.% PANI@NiTiO_3_ and 2.46 eV for 1wt.% PANI@CoTiO_3_, enhancing light absorption across UV and visible ranges. Scanning electron microscopy (SEM) and energy dispersive X-ray spectroscopy (EDS) analysis demonstrated the uniform distribution of PANI, ensuring consistent surface activity and efficient charge transfer. The photocatalytic test confirmed a pseudo-first-order degradation mechanism. The study elucidates the degradation mechanism through intermediate identification via HPLC-MS analysis, highlighting N-de-ethylation, aromatic ring cleavage and eventual mineralization into CO_2_ and H_2_O as critical pathways. Furthermore, the 1wt.%PANI@NiTiO_3_ nanocomposite demonstrated excellent stability and recyclability, maintaining its degradation efficiency over four consecutive cycles with minimal change. These findings highlight the potential of PANI@XTiO_3_ nanocomposites for sustainable and efficient wastewater treatment, addressing diverse environmental challenges by tailoring photocatalysts to specific light sources.

**Supplementary Information:**

The online version contains supplementary material available at 10.1038/s41598-024-83610-1.

## Introduction

In recent years, increasing urbanization and technical improvements in many industries have significantly increased the variety and quantity of contaminants in water bodies, resulting in severe water pollution problems^1^. Advances in water quality monitoring technologies allow the detection of micropollutants at low concentrations^2,3^. Due to the widespread contamination of water sources by industrial effluents, particularly those from the textile sector, the removal of synthetic organic dyes has become a critical environmental issue. While many methods—biological, ozonation, membrane filtration, physicochemical, and advanced oxidation processes are used to treat dye-laden effluents are often limited by variables such as extended operating times, low efficiencies, and excessive costs^4–6^.

Among the most recent technologies, photocatalytic degradation has attracted significant interest due to its highly efficient solar energy utilization to destroy organic contaminants. This process uses the high oxidizing properties of hydroxyl and superoxide radicals, resulting in rapid and effective water treatment^7,8^. The photocatalysis mechanism starts when the light (typically a lamp or sun) excites the photocatalyst, causing electrons in the valence band to move to the conduction band, creating electron-hole pairs^9^. These charge carriers interact with water and oxygen to produce hydroxyl radicals and superoxide anions that attack and break down organic pollutants like dyes, pharmaceuticals, and other emerging contaminants. The efficiency of photocatalysis depends on the photocatalyst’s ability to absorb light, separate the electron-hole pairs effectively and minimize recombination, which reduces the overall photocatalytic activity^10^ Despite the numerous benefits of this technology, including selectivity, high reaction rates, and ease of use, the primary challenge remains the development of highly efficient photocatalysts^11–14^.

Investigating photocatalytic materials for removing organic contaminants has constituted a significant research priority. Perovskites and perovskite-related structures offer diverse potential applications in developing novel photocatalysts for this process^15^. They are considered promising materials for the future development of renewable energy sources, energy storage solutions, and the degradation of pollutants due to their exceptional catalytic and photoelectric properties^16^. Perovskites are attracting considerable interest due to their unique electronic and crystal structures and notable photophysical and electrochemical properties^17^. Recent progress has contributed to developing calcium-titanium oxides such as NiTiO_3_ and CoTiO_3_, which exhibit photocatalytic properties^18,19^ Notably, perovskites have attracted considerable research due to several favourable properties, including a narrow energy bandgap, high carrier mobility, substantial adsorption coefficient, and overall stability^20,21^. However, the catalytic efficiency of pure perovskite is currently limited since its photogenerated electron-hole pairs recombine quickly, reducing its efficiency as a photocatalyst under visible light^22^. Research into using NiTiO_3_ and CoTiO_3_ has attracted significant attention due to their potential applications in environmental remediation. One study highlighted the synthesis of NiTiO_3_ and CoTiO_3_ nanoparticles, demonstrating that these catalysts effectively degraded Rhodamine B (RhB), achieving 30% degradation with CoTiO_3_ and 48% with NiTiO3in 3 h under a 35 W Xe lamp (3200 lumens, 6000 K) simulating sunlight^23^. Additionally, a Z-scheme NiTiO_3_/g-C_3_N_4_ catalyst performed even better, degrading RhB completely in 100 min under visible light^24^. Similarly, a NiTiO_3_/Bi_4_NbO_8_Cl showed high photocatalytic efficiency, degrading 90% of RhB within 5 h using a 300-W Xe lamp^25^. Other studies explored CoTiO_3_/BiOBr catalysts, which demonstrated enhanced photocatalytic activity for RhB degradation under visible light^26^, while CoTiO_3_/BiOI p-n photocatalysts achieved 100% degradation of RhB in just 60 min under visible light^20^.

The development of heterogeneous-conducting polymer nanocomposites, mainly organic/inorganic composites, has further expanded the potential applications of polyaniline. As research progresses, the integration of polyaniline with inorganic particles continues to offer promising solutions for advanced water treatment technologies, addressing the pressing need for effective and sustainable pollution remediation methods^27^. These materials enhance photo-induced charge separation and reduce charge recombination rates in electron transfer processes^28^. Polyaniline is distinguished by its distinctive electron and hole-transporting properties, simplicity of preparation, chemical stability, and environmental resilienc^29^. Studies have demonstrated that the integration of PANI with semiconductors enhances interfacial charge carrier separation, thereby augmenting photo-electrocatalytic activity. Furthermore, polyaniline’s high electrochemical activity, ease of protonation reversibility, excellent redox recyclability, and low cost collectively render it an attractive material for many applications^30,31,33^.

In this study, a novel nanocomposite of polyaniline@XTiO₃ (X = Ni and Co) with varying quantities of polyaniline (PANI) was synthesized via in-situ oxidative polymerization, and, for the first time, these materials have been employed in photocatalysis applications. A systematic investigation was conducted to elucidate the underlying mechanisms responsible for enhanced photocatalytic efficiency, encompassing comprehensive studies on the crystal structure, morphology, and optical properties using X-ray diffraction (XRD), Fourier transform infrared (FTIR) spectroscopy, UV–vis diffuse reflectance spectroscopy, and scanning electron microscopy with energy-dispersive X-ray spectroscopy (SEM–EDS). The photocatalytic performance of the nanocomposites was evaluated through the degradation of RhB, as a model of organic contaminants. The reusability of the photocatalyst was also investigated. The degradation pathway was determined by identifying intermediates using HPLC-MS.

## Materials and methods

### Synthesis of XTiO_3_

The perovskites were synthesized by a simple combustion method. (Co (NO_3_)_2_, 6H_2_ O), (Ni (NO_3_)_2_, 6H_2_O), TiO_2_-P25, and citric acid were used to synthesize perovskites. Initially, these materials were added to deionized water under vigorous magnetic stirring in a fixed molar ratio, specifically a 1:1:2 ratio of metal, TiO_2_, and citric acid, respectively. The resulting mixture evaporated at 80 to 130 °C to produce a thick gel. This gel was dried to convert it into dry form. Finally, the extracted gel was calcined at 700 °C for 4 h with a ramp of 10 °C per minute^32^.

### Synthesis of PANI@XTiO_3_

The PANI@XTiO_3_ nanocomposites were prepared through in-situ polymerization of aniline on XTiO_3_ perovskites. A fixed amount of XTiO_3_ perovskites was mixed in 100 ml of 1 M HCL for 30 min. After this, a specific amount of aniline was then added to the mixture and stirred for 1 h. Subsequently, 50 ml of FeCl_3_ (monomer/oxidant molar ratio set to 1:2) was added by drops into the reaction mixture. The polymerization reaction continued for 12 h under agitation at room temperature. The resulting product was thoroughly washed with deionized water and ethanol to eliminate any remaining oligoaniline and excess oxidant. Finally, the precipitation was maintained at 80 °C overnight. In this experimental process, various initial molar ratios of aniline (1wt.% and 2wt.%) were employed to synthesize XTiO_3_ coated with PANI. These nanocomposites are designated as 1wt.% PANI@NiTiO_3_, 2wt.% PANI@NiTiO_3_, 1wt.% PANI@CoTiO_3_, and 2wt.% PANI@CoTiO_3_.

### Characterization methods

The catalyst phases were identified through X-ray diffraction using a Schimaduz 6100 powder diffractometer equipped with a Cu Kα source. Identification of diffraction peaks adhered to the standards set by the Joint Committee on Powder Diffraction Standards (JCPDS). Fourier transform infrared spectroscopy (FTIR) with KBr (υ, in cm^− 1^) was employed to analyze the vibrational modes using the Nicolet iS50 instrument. The scans were conducted in transmission mode within the 4000 to 400 cm^− 1^ with a resolution of 4 cm^− 1^. Scanning Electron Microscopy (SEM) coupled with Energy-Dispersive Spectroscopy (EDS) performed the polycrystalline morphology and local composition by Quattros S-FEG-Thermofisher scientific instrument. The diffuse reflectance spectra (DRS) of the photocatalysts were recorded using a UV-vis spectrophotometer, Thermos Scientific Evolution 260 Bio, and the optical band gap energy was calculated using the Kubelka-Munk function^34^:

1 $$\:A(h\upsilon\:-{E}_{g})={\left(\alpha\:h\upsilon\:\right)}^{n}$$

Where, h denotes Planck’s constant, α signifies absorbance, υ represents light frequency, A stands for the proportional constant, and n equals 1/2 for indirect and 2 for direct transitions.

### Photocatalysis experiments

The photocatalytic efficiency of perovskites and PANI@XTiO_3_ was investigated for the photocatalytic degradation of RhB. An aqueous dye solution (50 ml) with an initial concentration of 5 mg/l was used in the experiments at room temperature in a photocatalytic reactor. The photocatalyst was added to the RhB solution at a concentration of 1 g/L. The solution was magnetically agitated throughout the reaction to ensure a well-mixed solution. Before turning on the light to start the photocatalytic reaction, the suspension was stirred in the dark for 30 min. The reaction system was illuminated by placing a 50-watt LED lamp on the top of the reactor, and in the case of the UV lamp, it was dispersed in the solution. At the end of the reaction, the catalyst particles were removed from the RhB solution through a 0.45-µm microfilter. The absorbance of RhB was measured at 553 nm using a spectrum UV-Vis spectrophotometer. The photodegraded liquid sample was collected for HPLC-MS analysis. HPLC-MS experiments were performed using an Ultimate 3000 system connected to an Exactive Plus mass detector. The gradient was 30/70 (water/methanol) at a flow rate of 1 ml/min.

## Results and discussion

### Photocatalysts characterization

#### X-ray diffraction

**Fig. 1:**
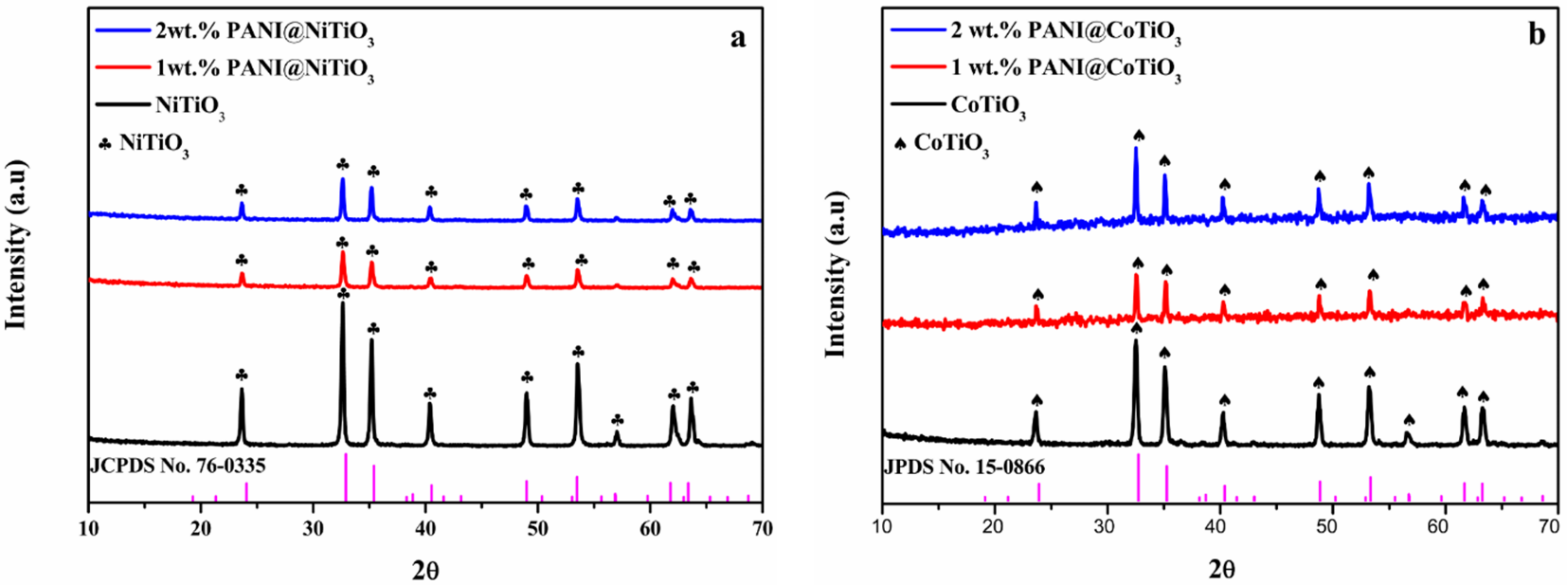
The DRX pattern of the prepared photocatalysts: (**a**) NiTiO_3_, and PANI@NiTiO_3_ nanocomposites, (**b**) CoTiO_3_, and PANI@CoTiO_3_ nanocomposites.

The X-ray diffraction (XRD) analysis of CoTiO_3_ and the PANI@CoTiO_3_ nanocomposites provided detailed insights into their structural characteristics, as illustrated in Fig. 1. Notably, the diffraction peaks were observed at specific 2θ angles at 23.73°, 32.57°, 35.15°, 40.29°, 48.74°, 53.37°, 61.71°, and 63.36°, referring to the (0 1 2), (1 0 4), (1 1 0), (1 1 3), (0 2 4), (1 1 6), (2 1 4), and (3 0 0) crystal planes of CoTiO_3_ as documented in JPDS No. 15-0866^19^. Interestingly, the XRD patterns of the PANI@CoTiO_3_ nanocomposites showed a remarkable similarity to those of pure CoTiO_3_ in both shape and peak position. This indicates that the PANI coating did not significantly affect the crystalline structure of CoTiO_3_. The similar diffraction patterns, along with the low content of PANI in the synthesized photocatalysts, explain why distinctive peaks of PANI are not observed in the composite patterns. Furthermore, the absence of additional peaks in the XRD patterns indicates the absence of impurities, thus affirming the high purity and successful synthesis of the CoTiO_3_ and PANI@CoTiO_3_ catalysts. The XRD analysis confirms that the structure of CoTiO_3_ is maintained even after the incorporation of PANI, suggesting that the PANI coating serves primarily to enhance the surface properties without disrupting the crystalline structure of CoTiO_3_, whose stability is important for the functional performance of these nanocomposites in practical applications.

In Fig. 1a, the X-ray diffraction (XRD) patterns of NiTiO_3_ and PANI@NiTiO_3_ nanocomposites are presented. Distinct diffraction peaks were observed at 2θ angles at 23.73, 32.57, 35.26, 40.40, 48.96, 53.52, 62.04, and 63.6°. These peaks correspond to the (0 1 2), (1 0 4), (1 1 0), (1 1 3), (0 2 4), (1 1 6), (2 1 4), and (3 0 0) crystal planes of NiTiO_3_, respectively, as documented in JCPDS card No. 76-0335^35^. Notably, the introduction of PANI did not cause any shifts in peak positions or alterations in peak shapes. Consequently, the XRD patterns of the PANI@NiTiO_3_ nanocomposites remained similar to those of pure NiTiO_3_. This suggests that the PANI coating did not affect the crystalline structure of NiTiO_3_. The consistent diffraction peaks at the specified angles indicate that the crystalline structure of NiTiO_3_ was preserved even after the incorporation of PANI. The absence of distinctive PANI peaks can be attributed to its low content and amorphous nature, which doesn’t produce sharp diffraction peaks detectable by XRD^36^. Furthermore, the absence of additional peaks indicates that the synthesized nanocomposites are free from impurities, confirming the high purity of the catalysts.

#### FT-IR spectroscopy

**Fig. 2:**
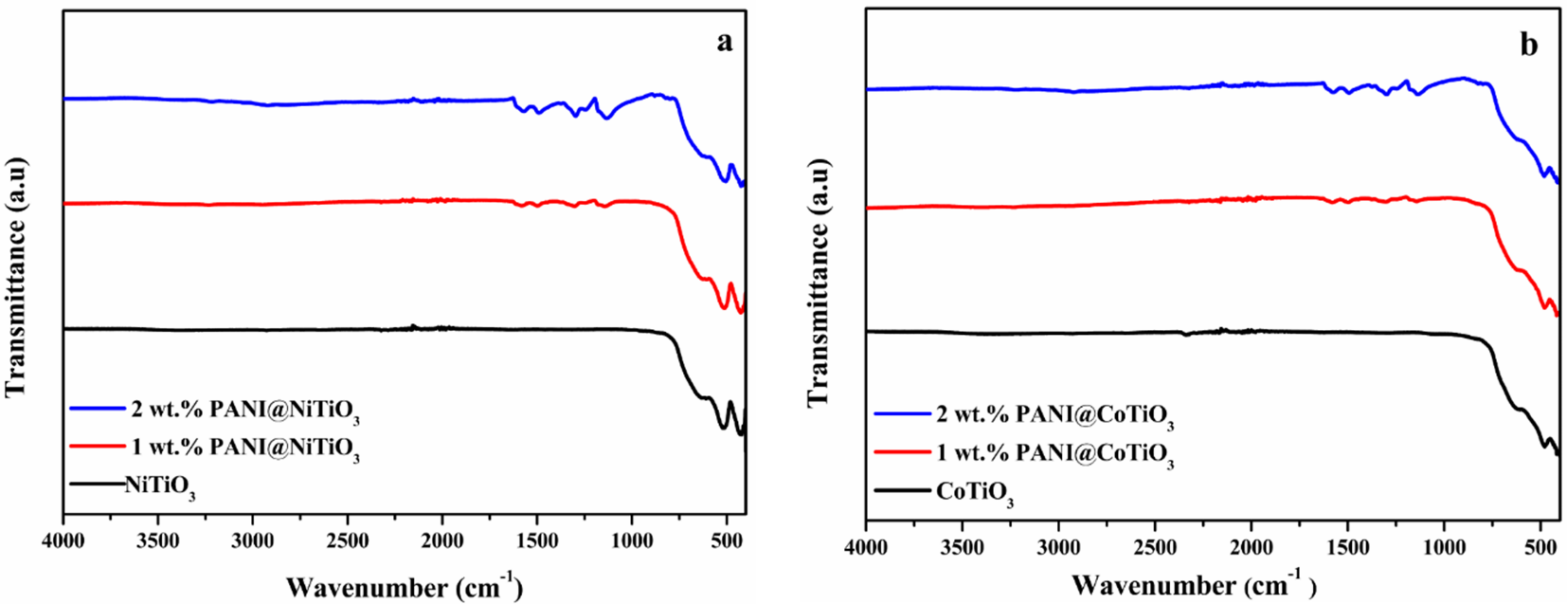
Infrared spectra of: (**a**) NiTiO_3_, and PANI@NiTiO_3_ nanocomposites, (**b**) CoTiO_3_, and PANI@CoTiO_3_ nanocomposites.

The FTIR spectroscopy results presented in Fig. 2a reveal distinct peaks associated with various functional groups for NiTiO_3_. In the pure perovskite spectrum, all the peaks are below 700 cm^− 1^. Simultaneously, the absorption peak at 424 cm^− 1^ is linked to the stretching of the Ni-O group, while the peaks at 515 and 631 cm^− 1^ are attributed to the Ti-O group^21^. Examining the FTIR spectrum of the nanocomposites, the peak around 3233 cm^− 1^ is associated with the N-H vibration caused by stretching of the PANI chain, and the band at 2940 cm^− 1^ corresponds to the C-H vibration of phenyl rings. Notably, the spectra exhibit characteristic bands at 1580 and 1490 cm^− 1^, signifying the C = C stretching vibration in quinoid benzenoid structures, and a band around 1300 cm^− 1^, indicating the C-N stretching vibration of aromatic amine. Additionally, peaks at about 1140 and 800 cm^− 1^ are associated with in-plane and out-of-plane deformation of C-H aromatic bonds in 1,4 di-substituted benzenes, respectively. The remaining peaks correspond to those characteristics of NiTiO_3_. In conclusion, the distinctive patterns observed in FTIR spectra strongly confirm the successful deposition of PANI on the NiTiO_3_ particles^37^.

The FTIR spectroscopy is provided in Fig. 2b. In the spectrum of CoTiO_3_, the broad peak observed at 403 cm^− 1^ is attributed to the stretching of the Co-O group. In addition, the absorption peaks at 475 and 618 cm^− 1^ can be assigned to the Ti-O group^38^. Furthermore, in the FTIR spectra of the PANI@CoTiO_3_ nanocomposites, the bond at 3228 cm^− 1^ is associated with the N-H stretching vibration of the PANI chain, while the peak at 2917 cm^− 1^ corresponds to the vibrational C-H bond in phenyl rings. Additionally, characteristic bands at 1580 and 1495 cm^− 1^ are assigned to C = C stretching vibrations in quinoid benzenoid structures. The band around 1300 cm^− 1^ is consistent with the vibrational stretching of the C-N of aromatic amine. The peaks at approximately 1135 and 818 cm^− 1^ are attributed to C-H aromatic in plane and out of plane deformation of 1,4 di-substituted benzene^39^. Therefore, these FTIR spectra provide the successful deposition of PANI on the CoTiO_3_ perovskite.

#### UV-VIS (DRS)

**Fig. 3:**
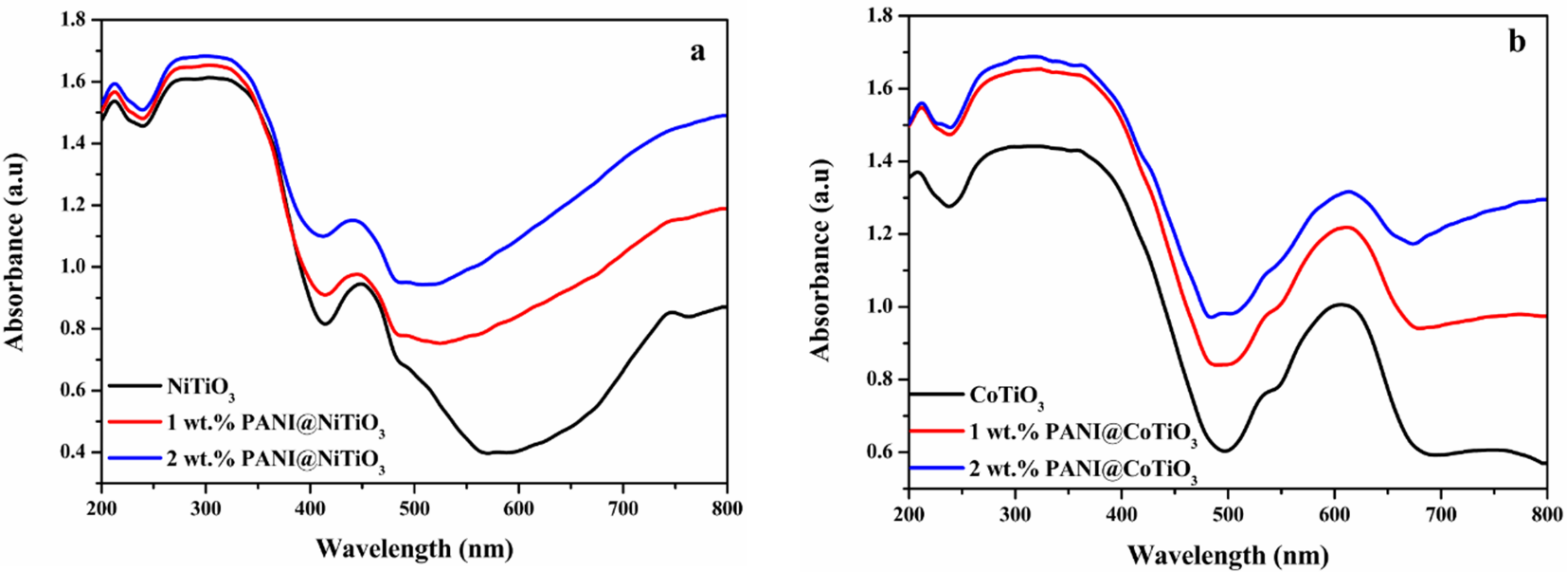
Diffuse reflectance spectra: (**a**) NiTiO_3_, and PANI@NiTiO_3_ nanocomposites, (**b**) CoTiO_3_, and PANI@CoTiO_3_ nanocomposites.

**Fig. 4:**
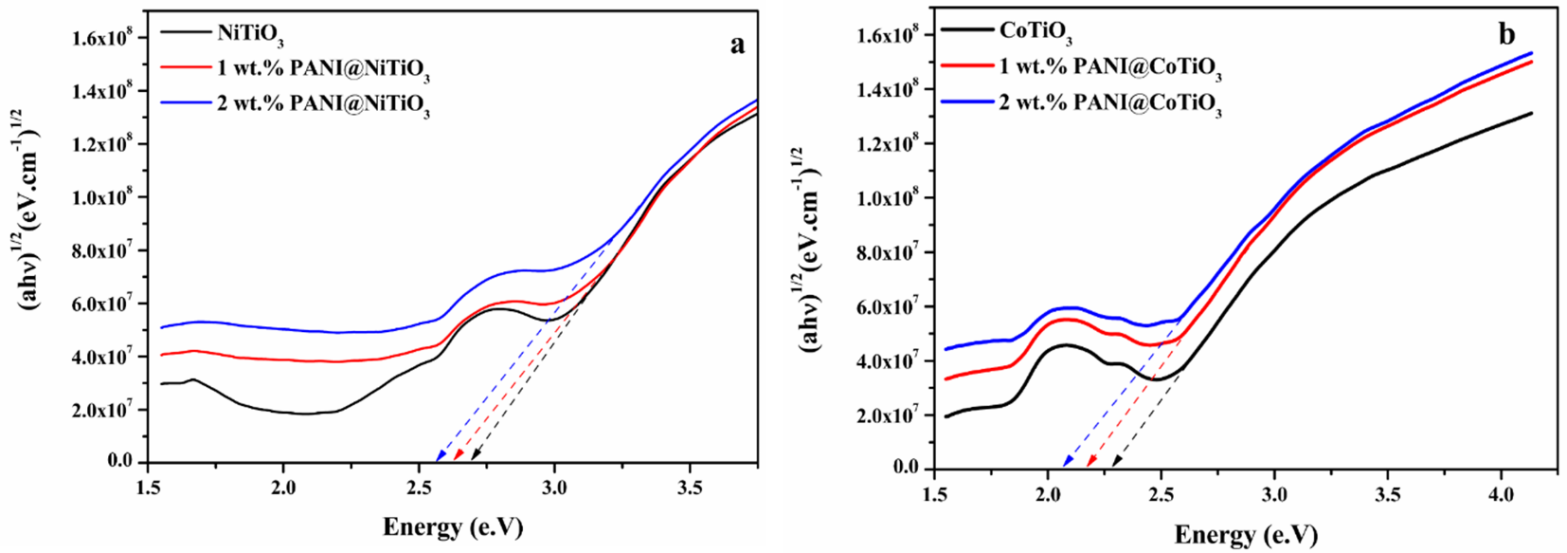
The Tauc plot of: (**a**) NiTiO_3_, and PANI@NiTiO_3_ nanocomposites, (**b**) CoTiO_3_, and PANI@CoTiO_3_ nanocomposites.

Figure 3a depicts the light absorption characteristics of NiTiO_3_ and PANI@NiTiO_3_ spanning the ultraviolet to visible light spectrum (200–800 nm). The perovskite’s and the nanocomposites’ UV-visible absorption spectra exhibit photoresponsivity across both the UV and visible light ranges. The broad peak in the UV region (at 318 nm) is ascribed to the charge transfer transition from the O^2−^-2p valence band to the Ti^4+^-3d conduction bands. Shifting to the visible region, two absorption bands appear at 450 nm and 510 nm, attributed to Ni-3d ↔Ti-3d and Ni-3d↔O-2p transitions, respectively^40^. Furthermore, the light absorption spectra display a substantial and broad absorption in the visible region, spanning from 600 to 800 nm, associated with the light yellow-coloured NiTiO_3_ perovskite. Consequently, adding PANI to NiTiO_3_ led to a modification in the absorption of the nanocomposites depending on the amount of PANI incorporated. With a 1wt.% PANI addition, the sample exhibited an intermediate alteration in absorption in the visible light region. Nevertheless, 2wt.% PANI@ NiTiO_3_ exhibits significantly improved absorption compared to NiTiO_3_ and 1wt.% PANI@NiTiO_3_, confirming the successful integration of PANI and NiTiO_3_. In Fig. 4a, the plot of (αhυ)^1/2^ vs. hυ is depicted. The band gap energy can be determined by drawing a tangent and identifying the intercept with the x-axis. The calculated band gap energies for NiTiO_3_, 1wt.% PANI@NiTiO_3,_ and 2wt.% PANI@NiTiO_3_ are approximately 2.70, 2.63, and 2.56 eV, respectively. The band gap classification obtained from the photocatalysts is as follows: NiTiO_3_ > 1wt.% PANI@NiTiO_3_ > 2wt.% PANI@NiTiO_3_. The narrower band gap observed in PANI@NiTiO_3_ nanocomposites compared to NiTiO_3_ is probably a result of the enhanced interaction within the hybrid structure, which enhances absorption in the solar spectrum. In addition, developing a conjugated structure between the π-conjugated structure of PANI and NiTiO_3_ enhances the effective migration of photo-induced charges while mitigating charge recombination. This contributes to enhanced photocatalytic performance in PANI@NiTiO_3_.

Figure 3b displays the optical absorption spectra of the CoTiO_3_ perovskite and PANI@CoTiO_3_ nanocomposites. The pure CoTiO_3_ demonstrates strong absorption over a broad spectrum, ranging from UV to visible light. The absorption peaks below 480 nm are associated with the charge transfer interaction between O^2−^ and Ti^4+^^41^. Furthermore, two absorption bands around 532 nm and 610 nm, linked with Co^2+^ Ti^4+^ charge transfer, is attributed to the crystal field splitting in CoTiO_3_^20^. In Fig. 4b, the graph illustrating (αhυ)^1/2^ versus hυ is presented. By using a tangent and identifying its intercept with the x-axis, the band gap of the photocatalysts can be determined. The estimated band gap energies of CoTiO_3_, 1wt.% PANI@CoTiO_3_, and 2wt.% PANI@CoTiO_3_ are approximately 2.88, 2.46, and 2.07 eV, respectively. The ranking of band gap classification among the photocatalysts is as follows: CoTiO_3_ > 1wt.% PANI@CoTiO_3_ > 2wt.% PANI@CoTiO_3_. The reduced band gap in PANI@CoTiO_3_ nanocomposites, in contrast to CoTiO_3_, is attributed to the robust interaction within the hybrid structure, which leads to improved solar spectrum absorption. Moreover, the formation of a conjugated structure between the π-conjugated structure of PANI and CoTiO_3_ enhances the efficient migration of photo-induced charges while minimizing charge recombination. This constructive interaction results in enhanced photocatalytic performance in PANI@CoTiO_3_.

#### SEM-EDS analysis

**Fig. 5:**
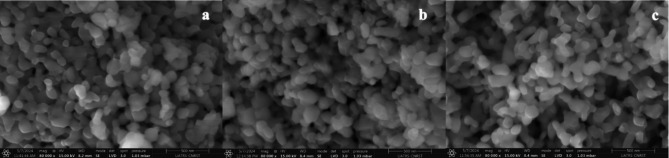
SEM image of: (**a**) CoTiO_3_, (**b**) 1wt.% PANI@CoTiO_3_, and (**c**) 2wt.% PANI@CoTiO_3_.

**Fig. 6:**
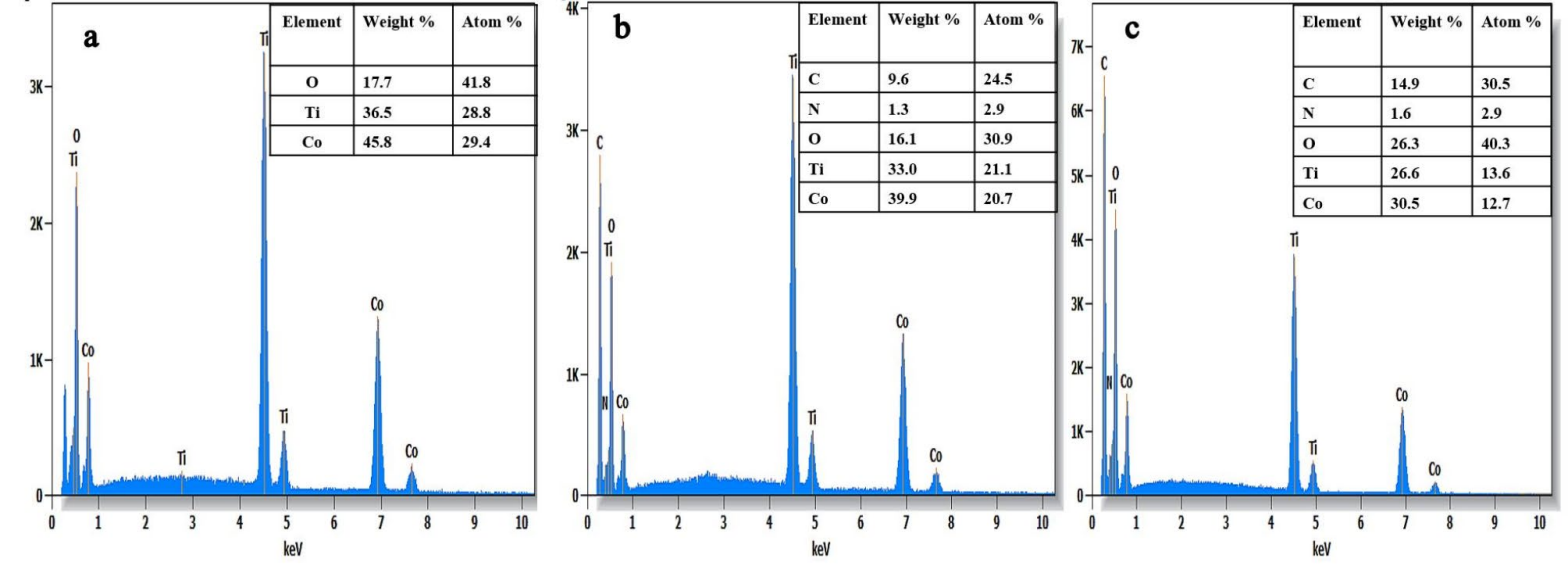
Energy dispersive X-ray spectra of: (**a**) CoTiO_3_, (**b**) 1wt.% PANI@CoTiO_3_, and (**c**) 2wt.% PANI@CoTiO_3_.

The morphology and surface characteristics of CoTiO_3_ perovskite were analyzed by scanning electron microscopy (SEM). The SEM micrographs (Fig. 5a) depict a homogeneous structure with agglomerated grains ranging from 100 to 400 nm. This aggregation and the weak porosity are attributed to the synthesis temperature. In addition, SEM images of the CoTiO_3_ catalyst revealed that the particles were uniformly small with minimal accumulation. Furthermore, SEM analysis provided detailed insight into the morphology of the particles, showing that they were uniformly spherical and evenly distributed across the sample, as illustrated in Fig. 5a. These particles have particle sizes in the range of 40–100 nm. To further investigate the chemical composition and purity of the CoTiO_3_, energy dispersive X-ray spectroscopy (EDS) analysis was performed. According to the EDS spectrum (Fig. 6), the CoTiO_3_ catalyst consists exclusively of the elements Co, Ti, and O. The absence of impurities in the EDX spectrum indicates that the synthesized CoTiO_3_ perovskite is pure and devoid of any surfactants or impurities.

**Fig. 7:**
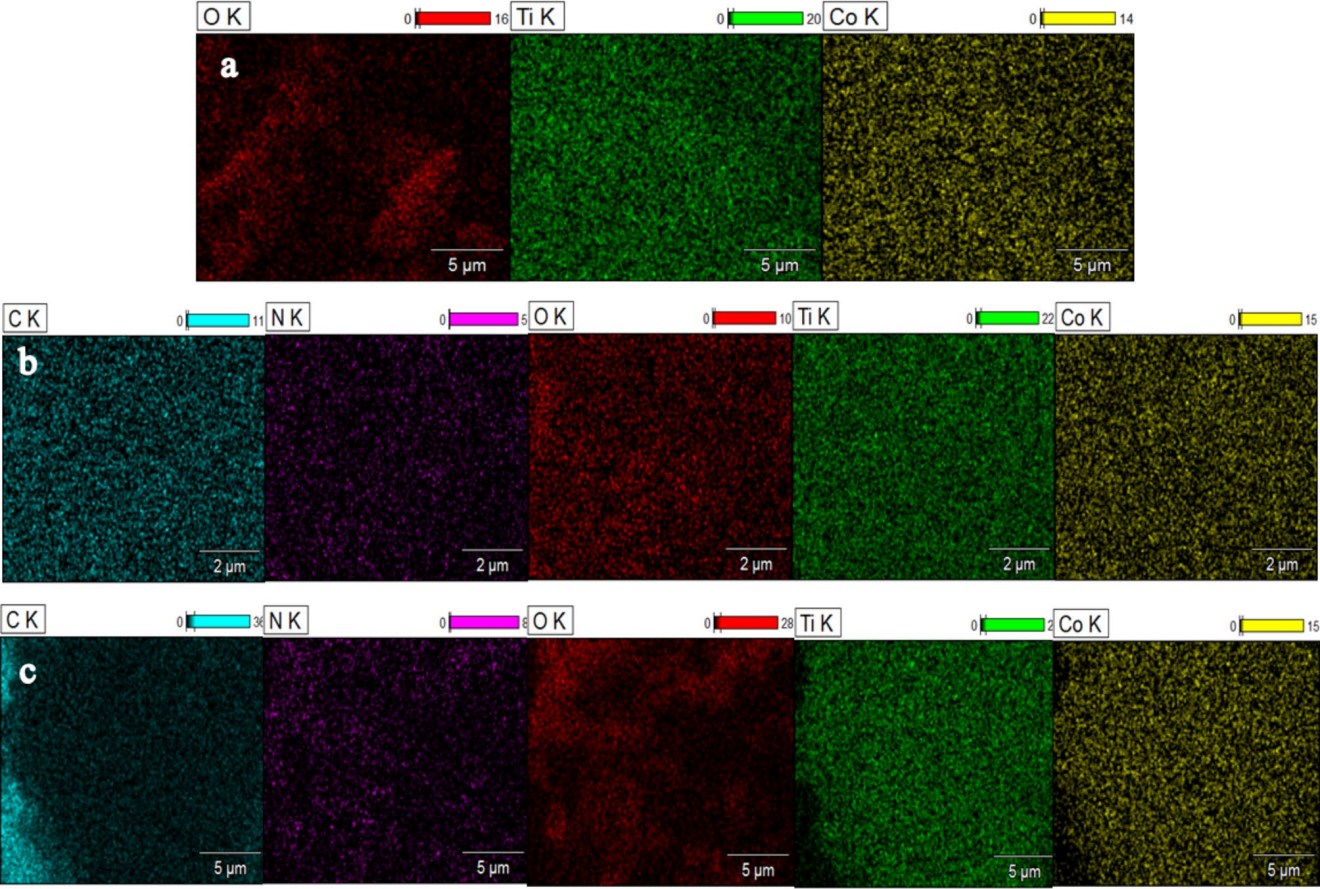
Energy dispersive X-ray mapping of: (**a**) CoTiO_3_, (**b**) 1wt.% PANI@CoTiO_3_, and (**c**) 2wt.% PANI@CoTiO_3_.

After incorporating PANI, the SEM micrograph of the PANI@CoTiO_3_ revealed small particles on the surface of CoTiO_3_, as seen in the SEM images (Fig. 5b and c). EDS elemental analysis of the catalyst samples, shown in Fig. 6a and b, and 6c, indicated that CoTiO_3_ contained Co, Ti, and O elements. In contrast, PANI@CoTiO_3_ nanocomposites comprised C, N, Co, Ti, and O. No additional impurity peaks were detected, confirming the high purity of the prepared PANI@CoTiO_3_ nanocomposites. SEM-EDS mapping provided a detailed analysis of the elemental distribution within the CoTiO_3_ and PANI@CoTiO_3_ nanocomposites (Fig. 7). The mapping images demonstrated a uniform distribution of Co, Ti, and O elements throughout the CoTiO_3_ particles, as evidenced by the consistent and even spread of these elements throughout the sample. In the case of the PANI@CoTiO_3_ nanocomposites, SEM-EDS mapping also revealed the presence of additional elements associated with the PANI coating, specifically N and C alongside the Co, Ti, and O already identified in the CoTiO_3_ structure. This uniform distribution indicates that the PANI has successfully and evenly polymerized onto the CoTiO_3_ to form a well-integrated nanocomposite material. The clarity of the EDS spectrum and the absence of any significant elemental segregation or clustering in the mapping images confirm the homogeneity and purity of both the CoTiO_3_ and PANI@CoTiO_3_ nanocomposites, underscoring the effectiveness of the synthesis process.

**Fig. 8:**
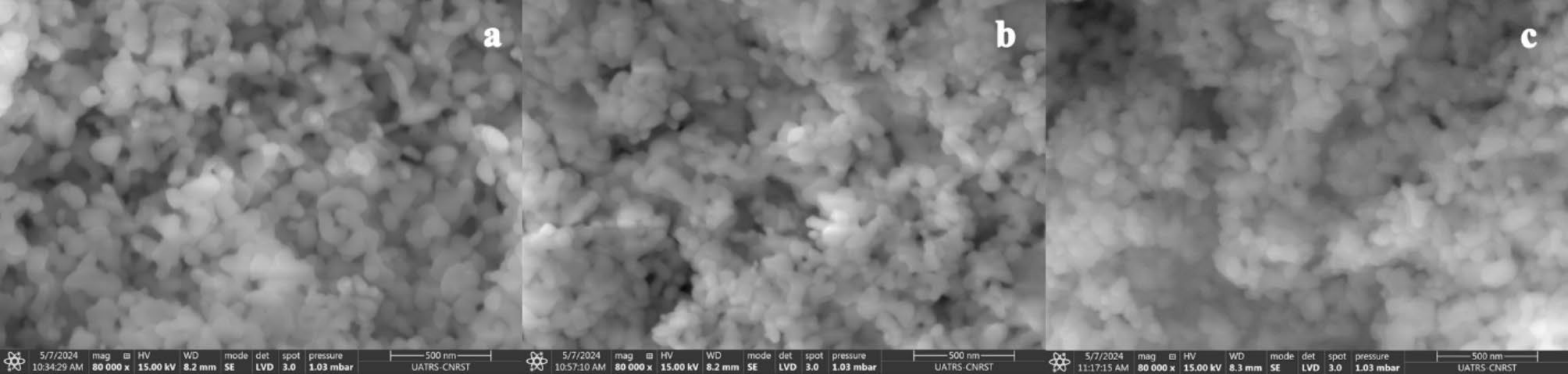
SEM image of: (**a**) NiTiO_3_, (**b**) 1wt.% PANI@NiTiO_3_, and (**c**) 2wt.% PANI@NiTiO_3_.

**Fig. 9:**
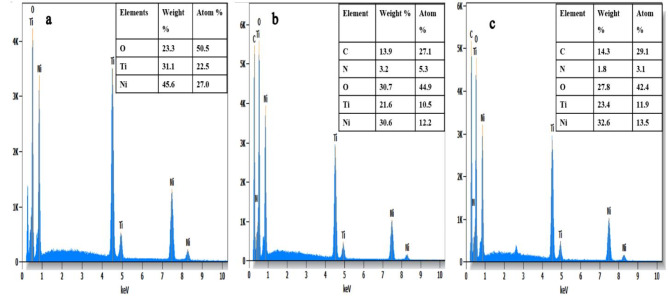
Energy dispersive X-ray spectra of: (**a**) NiTiO_3_, (**b**) 1wt.% PANI@NiTiO_3_, and (**c**) 2wt.% PANI@NiTiO_3_.

The SEM micrographs (Fig. 8a) of NiTiO_3_ showed a homogeneous structure with agglomerated grains ranging from 100 to 300 nm and low porosity. SEM images of NiTiO_3_ catalysts revealed particles with negligible agglomeration ranging from 30 to 100 nm. Furthermore, SEM showed detailed information about the shape of the particles, indicating that they were all identical in size and shape and evenly distributed throughout the sample (Fig. 8b). The smallest of these particles was about 30–40 nm, as shown in Fig. 8c. To investigate the chemical composition and purity of the NiTiO_3_ perovskite, energy-dispersive X-ray spectroscopy (EDS) was used. The EDS spectrum (Fig. 9a) indicated that the catalyst was entirely composed of nickel (Ni), titanium (Ti), and oxygen (O). The absence of contaminants in the EDS results confirms the purity of the synthesized NiTiO_3_ perovskite and affirms the effectiveness of the synthesis technique in creating high-purity perovskite. In contrast to other nanocomposites, the PANI@NiTiO_3_ nanocomposite (Fig. 9b) showed the presence of carbon (C) and nitrogen (N) elements in addition to Ni, Ti, and O. The absence of impurity peaks indicates the purity of the PANI@NiTiO_3_ nanocomposites (Fig. 9c).

**Fig. 10:**
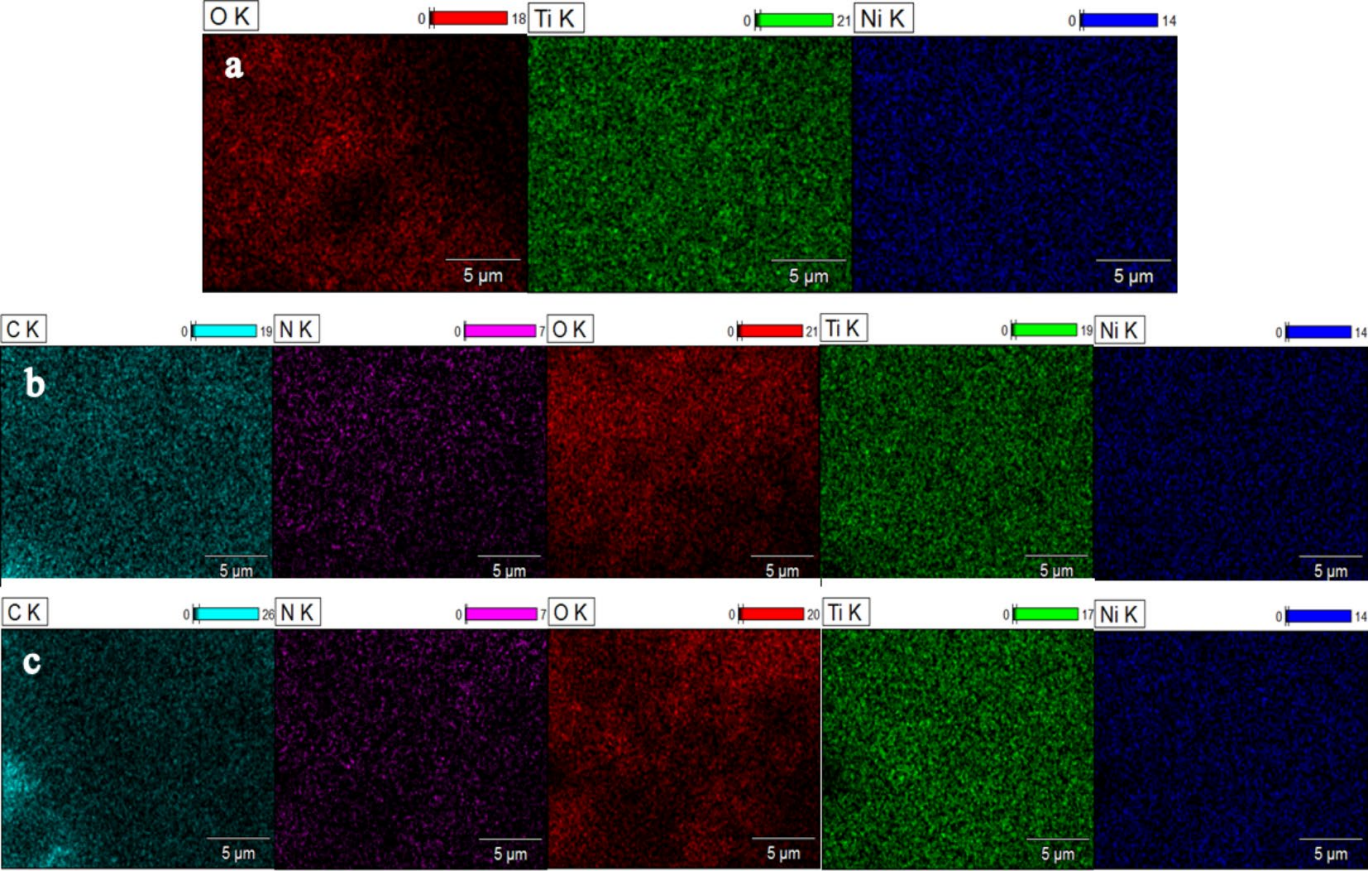
Energy dispersive X-ray mapping of: (**a**) NiTiO_3_, (**b**) 1wt.% PANI@NiTiO_3_, and (**c**) 2wt.% PANI@NiTiO_3_.

SEM-EDS mapping allowed a comprehensive analysis of the elemental distribution in the NiTiO_3_ and PANI@NiTiO_3_ nanocomposites. The mapping images (Fig. 10a) showed a consistent and uniform distribution of the nickel (Ni), titanium (Ti), and oxygen (O) elements across the NiTiO_3_. This indicates that these elements are uniformly dispersed throughout the sample. In addition, the SEM-EDS mapping of PANI@NiTiO_3_ nanocomposites (Fig. 10b and c) revealed the existence of nitrogen (N) and carbon (C) elements, which were attributed to the PANI coating. These elements were found alongside the already identified Ni, Ti, and O elements within the NiTiO_3_ structure. The uniform distribution indicates that the PANI has successfully polymerized onto the NiTiO_3_, resulting in a well-integrated composite. In the mapping images, there is no noticeable elemental segregation or clustering. This confirms that the NiTiO_3_ and PANI@NiTiO_3_ nanocomposites are homogeneous and pure, highlighting the success of the synthesis process.

### Photodegradation of Rhodamine B

**Fig. 11:**
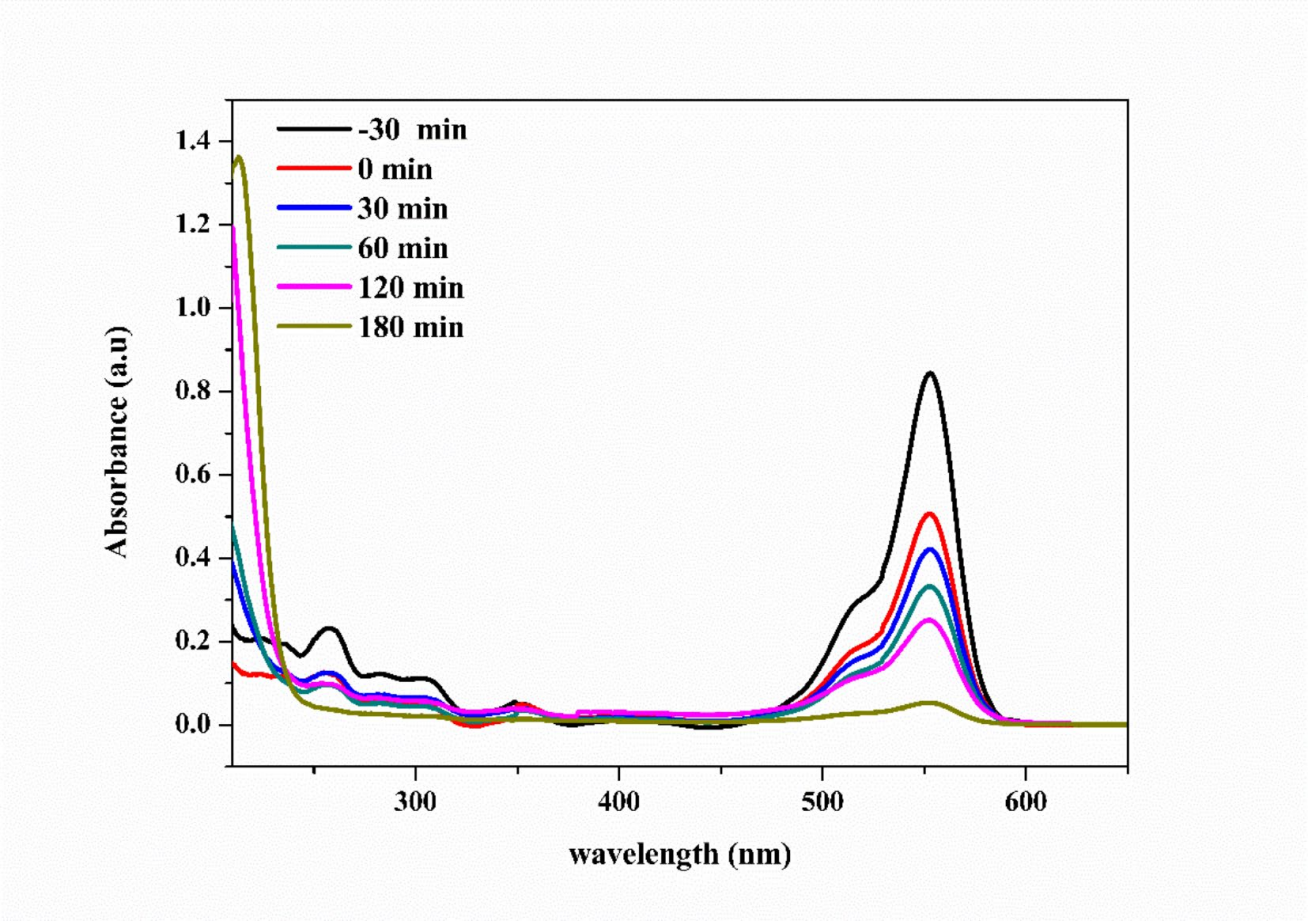
UV-Vis spectrum of RhB photodegradation with 1wt.% PANI@NiTiO_3_ photocatalyst using LED lamp.

The photocatalytic activity of the synthesized photocatalysts was evaluated by monitoring the degradation of RhB in an aqueous solution under the irradiation of a 50 W LED lamp and UV lamp (λ = 254 nm) at room temperature. Figure 11 shows the degradation spectra of RhB.

In RhB photodegradation, reactive oxygen species (ROS) play a crucial role. Under light irradiation, PANI@XTiO_3_ nanocomposites generate electrons (e^−^) and holes (h^+^) on their surface (Eq. (2)). These charges interact with water and oxygen molecules to form hydroxyl radicals ($\:{HO}^{^\circ\:}$) and superoxide ions ($\:{O}_{2}^{^\circ\:-}$) as demonstrated in Eqs. (3)-(6), which then attack RhB molecules, causing N-De-ethylation and opening of aromatic rings, leading to their mineralization into CO_2_ and H_2_O (Eq. (7)).

2 $$\:{PANI@XTiO}_{3}+h\upsilon\:\to\:{PANI@XTiO}_{3}({e}^{-}+{h}^{+})$$

3 $$\:{O}_{2}+{e}^{-}\to\:{O}_{2}^{^\circ\:-}$$

4 $$\:{O}_{2}^{^\circ\:-}+{H}^{+}\to\:{HOO}^{^\circ\:}$$

5 $$\:{2HOO}^{^\circ\:}\to\:{H}_{2}{O}_{2}+{O}_{2}$$

6 $$\:{H}_{2}O+{h}^{+}\to\:{HO}^{^\circ\:}+{H}^{+}$$

7 $$\:RhB\left(Dye\:molecule\right)+\left({HO}^{^\circ\:},{HOO}^{^\circ\:},{H}^{+}or{H}_{2}{O}_{2}\right)\to\:Intermediate\:products\to\:{CO}_{2}+{H}_{2}O$$

#### Photodegradation of Rhodamine B with LED lamp

**Fig. 12:**
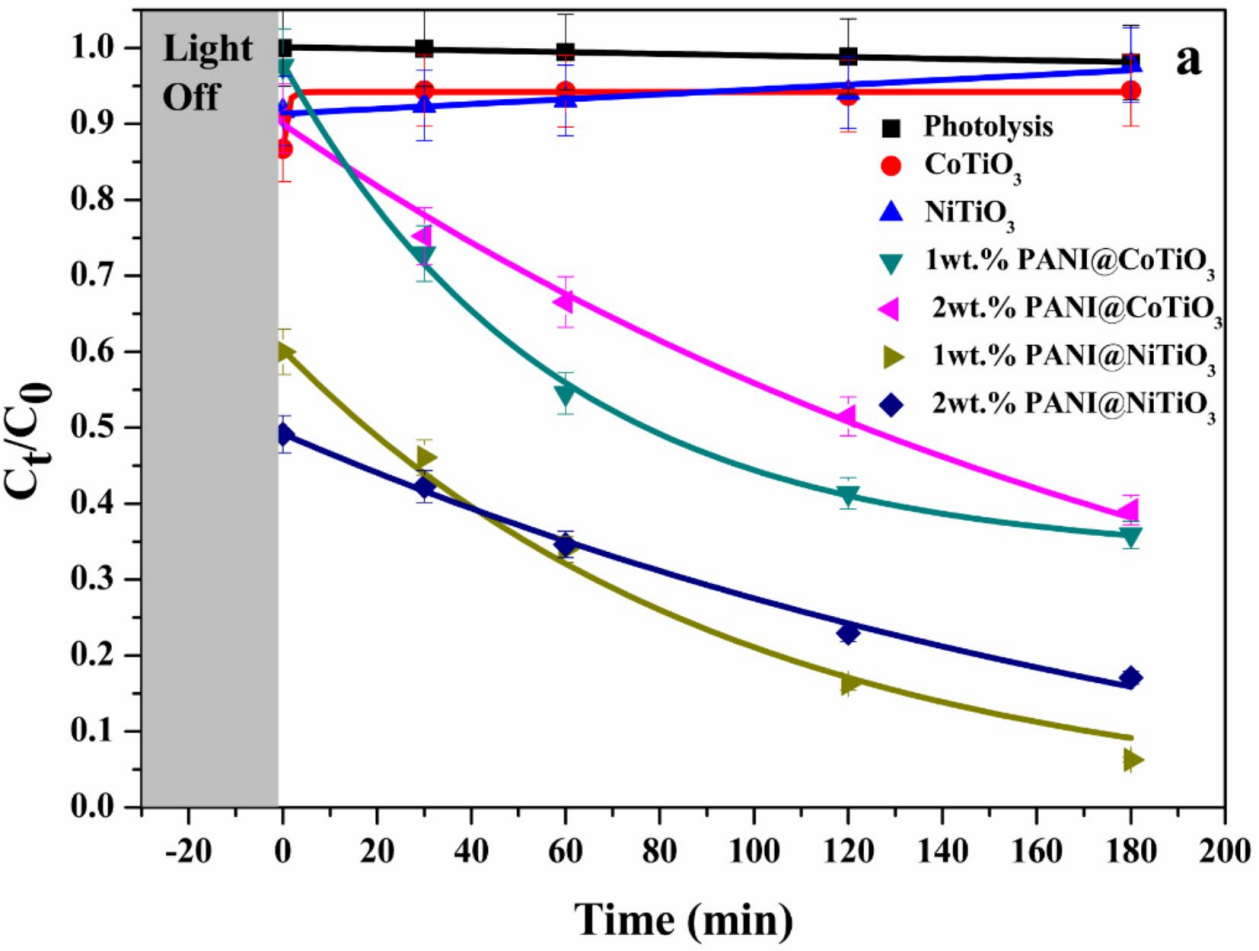
Photodegradation of RhB over photocatalysts under LED lamp irradiation; Lines show the corresponding first order kinetic fits.

**Fig. 13:**
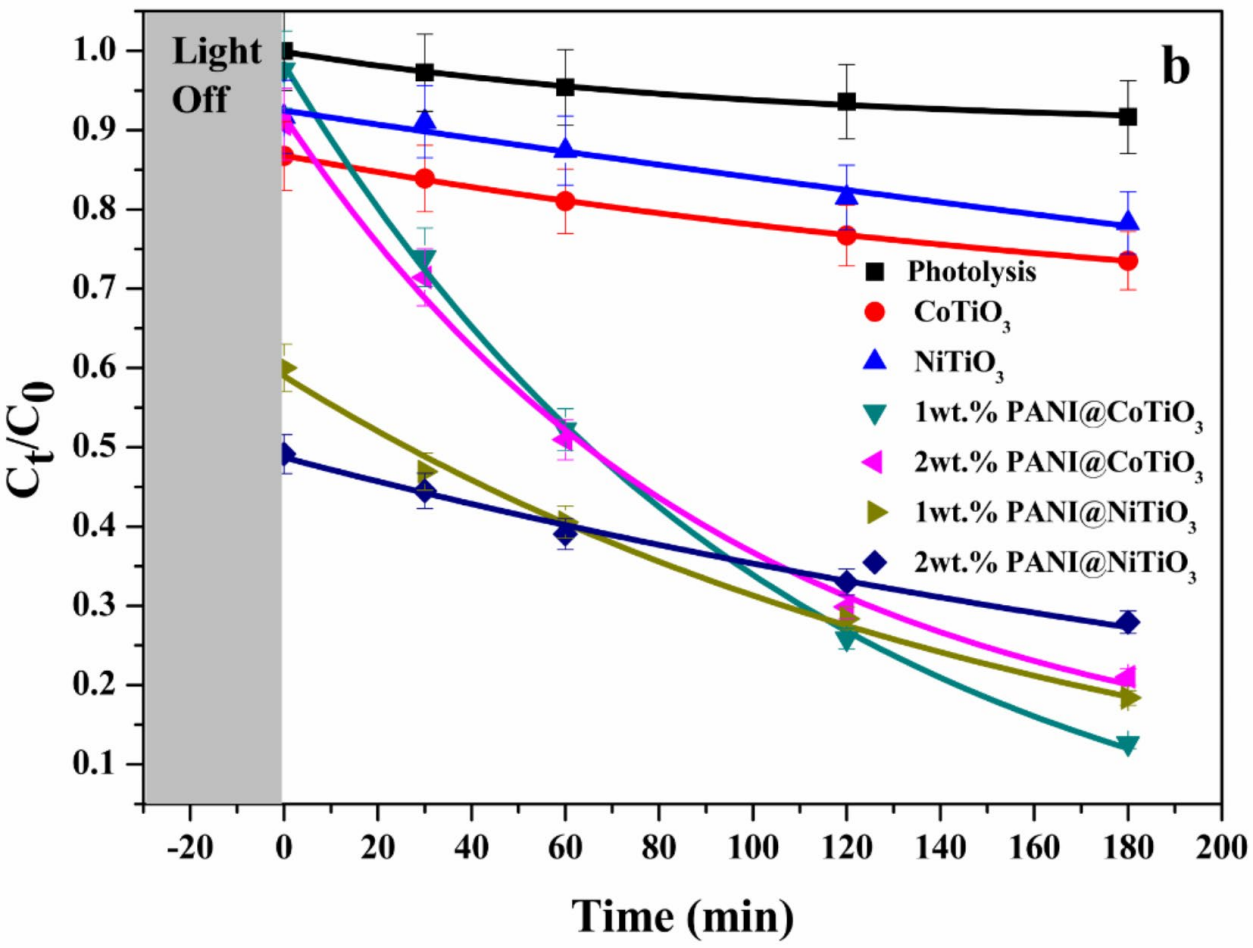
Photodegradation of RhB over photocatalysts under UV lamp irradiation; Lines show the corresponding first order kinetic fits.

The experimental data accurately fit a first-order kinetic model. Figures 12 and 13 show the photodegradation efficiency with and without the photocatalyst. After 180 min of photocatalysis, the initial results indicated that, in the absence of photocatalysts, the photodegradation was negligible, demonstrating the stability of RhB under UV light and LED irradiation. Under LED light irradiation (Fig. 12), the degradation of RhB was less than 10% after approximately 180 min when using CoTiO_3_ and NiTiO_3_, respectively. This low photodegradation efficiency highlighted the need for enhanced photocatalysts. Incorporating polyaniline (PANI) into CoTiO_3_ and NiTiO_3_ significantly improved photocatalytic efficiency. Specifically, the photodegradation efficiencies for 1wt.% PANI@NiTiO_3_, 2wt.% PANI@NiTiO_3_, 1wt.% PANI@CoTiO_3_, and 2wt.% PANI@CoTiO_3_ were 94, 83, 64, and 61%, respectively. The highest photodegradation was achieved with 1wt.% PANI@NiTiO_3_ composite, which reached 94% degradation of RhB within 180 min. These findings highlight the critical role of PANI in enhancing photocatalytic activity.

The data indicates that the amount of PANI is crucial in determining photocatalytic degradation efficiency. PANI concentration enhances photocatalytic activity by improving the dispersion within the perovskite, which is essential for effective charge transfer and separation while maintaining surface accessibility for oxidation processes. Kinetic analysis of RhB photodegradation in the presence of the PANI@NiTiO_3_ nanocomposite revealed a pseudo-first-order reaction mechanism. The apparent rate constant (k) for different PANI concentrations was calculated and presented in Table 1. The highest rate constant was 172.9 ± 25.5 × 10^4^ (min^− 1^) for 1wt% PANI@CoTiO_3_ nanocomposites; this significant increase in the rate constant demonstrates that the optimal amount of PANI enhances the photocatalytic activity by facilitating better charge separation and transfer efficiency.

The study highlights that incorporating PANI into XTiO_3_ enhances the photocatalytic degradation of organic pollutants such as RhB under LED light irradiation. The optimal concentration of PANI is crucial for maximizing photocatalytic efficiency, as it promotes better dispersion and surface activity. However, excessive PANI can impede the process by obstructing reactive sites. Thus, finding the right balance in PANI concentration is essential for achieving superior photocatalytic performance.

**Table 1:** First-order rate constants obtained for RhB removal with photocatalysts under LED lamp irradiation.

Photocatalysts	(k ± σk). · 10^4^ (min^-1^)
1wt.% PANI@CoTiO_3_	172.9 ± 25.5
2wt.% PANI@CoTiO_3_	47.6 ± 1.606
1wt.% PANI@NiTiO_3_	105.3 ± 14.5
2wt.% PANI@NiTiO_3_	60.9 ± 16.3

#### Photodegradation of Rhodamine B with UV lamp

On the other side, under the UV light irradiation (Fig. 13), the RhB removal after 180 min was below 25% with CoTiO_3_ and NiTiO_3_. The addition of PANI to CoTiO_3_ and NiTiO_3_ increases their photocatalytic effectiveness. The photodegradation for 1wt.% PANI@CoTiO_3_, 2wt.% PANI@CoTiO_3_, 1wt.% PANI@NiTiO_3_, and 2 wt% PANI@NiTiO_3_ were 87, 79, 82, and 72%, respectively. The estimated rate constants (k) for various concentrations of PANI are displayed in Table 2. The rate constant for the 1wt.% PANI@CoTiO_3_ nanocomposite was 109.8 ± 9.08 × 10^4^ (min^− 1^).

The difference in the UV and visible light results can be attributed to the distinct light absorption characteristics of PANI@CoTiO_3_ and PANI@NiTiO_3_. The 1wt.% PANI@NiTiO_3_ nanocomposite demonstrated superior photocatalytic performance under LED light irradiation, effectively degrading RhB due to its ability to absorb and utilize the energy from the visible spectrum more efficiently. This can be linked to its electronic structure and band gap, which are well matched with the energy levels of visible light photons, thereby promoting effective electron excitation and subsequent photocatalytic reactions. On the other hand, 1wt.% PANI@CoTiO_3_ showed remarkable efficiency under UV light irradiation. The UV light, with its higher energy photons, aligns well with the band gap of CoTiO_3_, facilitating a higher rate of electron excitation and generation of reactive species necessary for the degradation of RhB. This efficiency under UV light can be attributed to the strong absorption of UV photons, which provide sufficient energy to overcome the band gap of CoTiO_3_ and initiate the photocatalytic process.

The study indicates that NiTiO_3_ is more suitable for applications requiring visible light activation, while CoTiO_3_ is more effective under UV light conditions. This highlights the importance of tailoring the choice of photocatalyst based on the specific irradiation conditions to optimize RhB’s degradation efficiency. The results emphasize the need to understand the interaction between light absorption and properties and photocatalytic performance for the development of more effective photocatalytic systems. For a comprehensive comparison, Table 3 presents the photocatalytic efficiencies of various photocatalysts for RhB degradation as documented in the literature.

**Table 2:** First-order rate constants obtained for RhB removal with photocatalysts under UV lamp irradiation.

Photocatalysts	(k ± σk) · 10^4^ (min^-1^)
1wt.% PANI@CoTiO_3_	109.8 ± 9.08
2wt.% PANI@CoTiO_3_	106.6 ± 18.5
1wt.% PANI@NiTiO_3_	60.6 ± 4.46
2wt.% PANI@NiTiO_3_	31.6 ± 2.01

**Table 3:** Comparison of photocatalytic degradation of RhB dye with the previous reports.

Catalysts	Light source	Catalyst amount	Time(min)	Degradation (%)	References
Fe^3+^ doped Bi_4_Ti_3_O_12_	Sunlight	1	180	97.11	^42^
BaTiO_3_/Au-2	250 W Hg lamp	0.4	180	76	^43^
Eu_2_O_3_	Xenon lamp (500 W)	1	60	22	^44^
FeS_2_/TiO_2_	Xenon lamp (300 W)	1	180	97	^45^
CuO-ZnO (10%)	Xenon lamp (100 W)	1.4	180	90.25	^46^
NiTiO_3_	35 W Xe lamp (3200 lumens, 6000 K)	1	180	48	^23^
CoTiO_3_	36 W Xe lamp (3200 lumens, 6000 K)	1	180	30	^23^
NiTiO_3_/Bi_4_NbO_8_Cl	Xe lamp (300 W)	1	300	90	^25^
CoTiO_3_/BiOBr	High-pressure xenon lamp (500 W)	1	50	100	^26^
PANI@NiTiO_3_	LED lamp (50 W)	1	180	94	This work
PANI@CoTiO_3_	UV lamp (7 W-254 nm)	1	180	87	This work

#### Photocatalytic degradation pathway

**Fig. 14:**
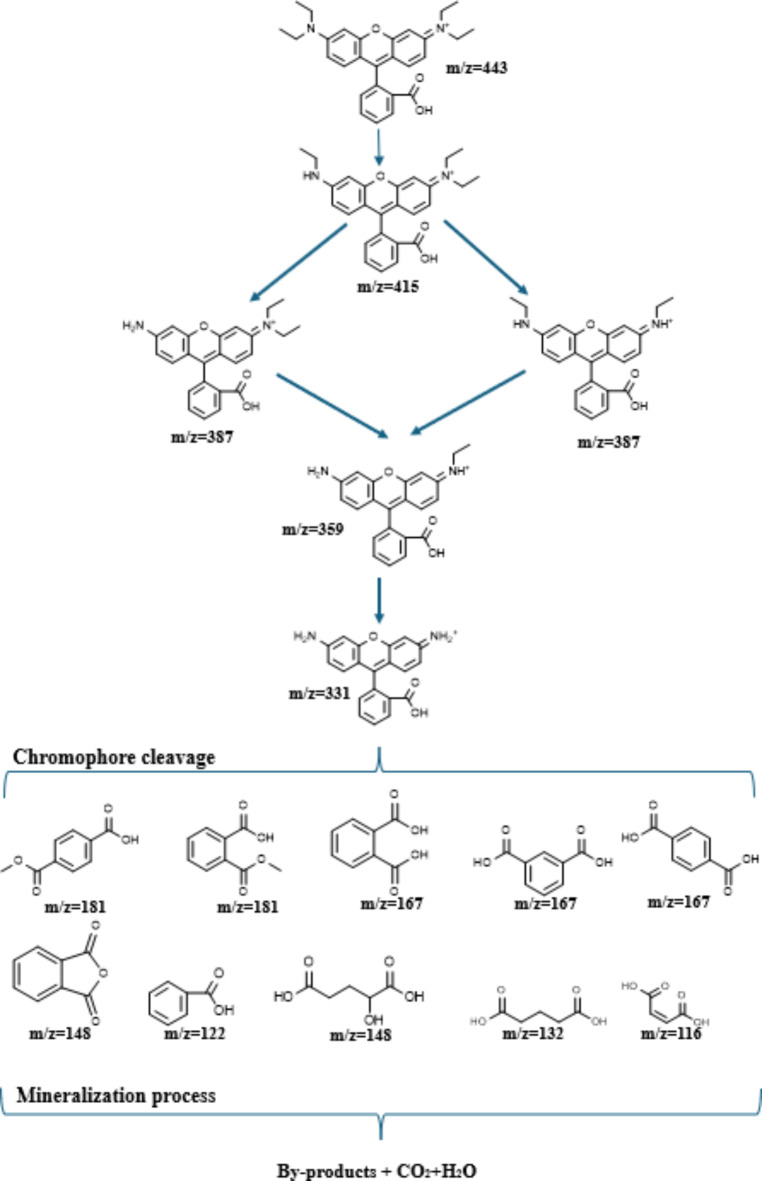
Proposed mechanisms for the photocatalytic degradation of RhB.

Identifying potential intermediate products during the photocatalytic reaction is crucial for understanding the mechanism of photocatalytic degradation. To investigate the degradation pathway of RhB dye under light irradiation and to identify the possible intermediates products, the main intermediates formed during the degradation process were proposed based on the m/z values obtained from the mass spectra (Figure S1). Figure 14 represents the possible degradation pathway of RhB.

The HPLC-MS analysis revealed the formation of several aromatic intermediates. In the presence of PANI@NiTiO_3_, a significant degradation of RhB was observed under LED lamp irradiation. According to previous studies^47–49^, most of the N-de-ethylation process occurred through the formation of nitrogen-centered radicals during the degradation of the RhB chromophore structure. The photocatalytic degradation of RhB was facilitated by photogenerated active species, such as hydroxyl radicals and photogenerated holes, which attacked the central carbon of RhB, causing decolorization of the dye, followed by degradation via N-de-ethylation. From the mass spectra, the central intermediates were identified based on m/z values of 443, 415, 387, and 359, corresponding to RhB, N-de-ethylated intermediates such as N, N-diethyl-N’-ethylrhodamine, N,N-diethylrhodamine, N-ethyl-N’-ethylrhodamine, and N-ethylrhodamine, respectively. The degradation of N-de-ethylated intermediates resulted in a photoproduct with an m/z value of 331, which underwent chromophore cleavage, followed by ring-opening reactions and eventual mineralization^50^. During these reactions, various intermediates were observed, including 4-(Methoxycarbonyl) benzoic acid, 2-(methoxycarbonyl) benzoic acid, phthalic acid, isophthalic acid, terephthalic acid, phthalic anhydride, 2-hydroxypentanedioic acid, glutaric acid, benzoic acid, and maleic acid, with m/z values of 181, 167, 148, 132, 122, and 116, respectively ( Figure. S1). Eventually, smaller molecules were degraded into CO_2_ and H_2_O. These results demonstrate that RhB can be effectively decomposed through photocatalytic reactions and transformed into nearly harmless substances.

#### Recyclability of the photocatalyst

**Fig. 15:**
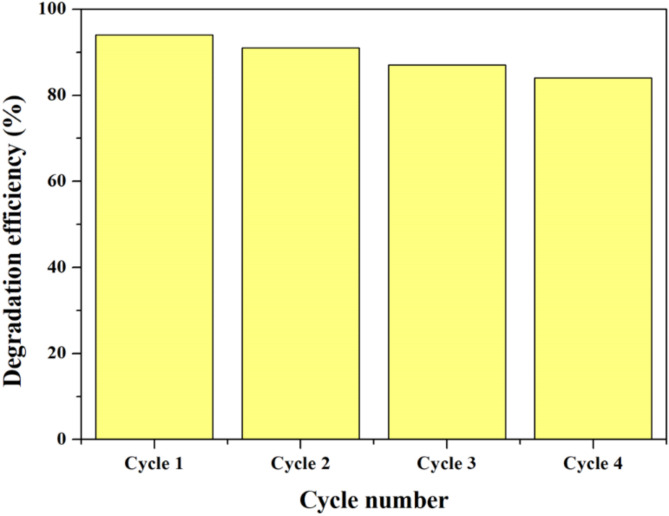
Effect of several runs on the photodegradation efficiency of RhB by 1wt.% PANI@NiTiO_3_.

The stability performance of 1wt.% PANI@NiTiO₃ nanocomposite was evaluated over four consecutive cycles for Rhodamine B (RhB) degradation of (Fig. 15). The results show that 1wt.%PANI@NiTiO₃ (under LED lamp) maintains a degradation efficiency of 84% after four cycles, starting from 94% in the first cycle. This gradual decrease is mainly attributed to a slight saturation of the active sites and potential material losses during recovery. These results confirm the chemical and structural stability of the nanocomposite, with high overall performances (> 84%) after several cycles. This stability highlights its potential for repeated applications in treating wastewater contaminated by organic pollutants.

## Conclusion

In conclusion, CoTiO_3_ and NiTiO_3_ perovskites were prepared by a combustion method followed by calcination and then polymerized with aniline to form nanocomposites with different percentages of PANI (1wt.% PANI@NiTiO_3_, 2wt.% PANI@NiTiO_3_, 1wt.% PANI@CoTiO_3_, and 2wt.% PANI@NiTiO_3_). The photocatalytic efficiencies of the perovskites and PANI@XTiO_3_ nanocomposites were evaluated for the photocatalytic degradation of RhB. X-ray diffraction showed the successful synthesis of XTiO_3_ perovskites, and the PANI did not affect the crystal structure of NiTiO_3_ and CoTiO_3_, ensuring the purity of the materials. FTIR spectra also confirmed the successful deposition of PANI onto XTiO_3_ perovskites. UV-vis DRS revealed changes in the optical properties of the materials, including significant optical absorption due to the interaction between PANI and the perovskite particles. SEM micrographs showed uniform particles with homogeneous element distribution. EDS analysis confirmed the pure composition of perovskites, consisting only of Ni, Ti, and O for NiTiO_3_ and Co, Ti, and O for CoTiO_3_. Adding PANI uniformly introduced C and N without impurities. SEM-EDS mapping revealed a uniform distribution of elements. The photocatalytic activity of the photocatalysts was performed to degrade RhB under LED and UV lamp irradiation. The results showed that without photocatalysts, the photodegradation was negligible, confirming the stability of RhB under these conditions. The addition of polyaniline (PANI) significantly improved the photocatalytic efficiency of the nanocomposites, reaching up to 94% degradation for 1wt.% PANI@NiTiO_3_ under LED light. The analysis revealed that the optimal concentration of PANI is crucial for maximizing photocatalytic efficiency by improving dispersion and surface activity while avoiding the clogging of reactive sites. Under UV light, the 1wt.% PANI@CoTiO_3_ nanocomposite achieved up to 87% removal of RhB. NiTiO_3_ performs better under visible light, whereas CoTiO_3_ is more efficient under UV light. This underscores the importance of selecting the appropriate photocatalyst based on irradiation conditions. The HPLC-MS analysis identified several aromatic intermediates during the photocatalytic degradation of RhB with N-de-ethylation being a a critical step. The degradation pathway included ring cleavage and mineralization, ultimately converting RhB into harmless products like CO_2_ and H_2_O. Additionally, the reusability test of 1wt.%PANI@NiTiO_3_ confirmed its remarkable stability, retaining an 84% degradation efficiency after four consecutive catalytic cycles. In future studies, these materials could be adapted for the degradation of other organic pollutants and integrated into industrial wastewater treatment processes. Further optimization of reaction parameters and exploration of other hybrid structures are needed to extend their applicability.

## Electronic Supplementary Material

Below is the link to the electronic supplementary material.

Supplementary Material 1

## Data Availability

The data presented in this study are available upon request from the corresponding author.
