# Supplementary figures and images for: Enhanced photocatalytic degradation of Rhodamine B using polyaniline-coated XTiO3(X = Co, Ni) nanocomposites
Source: Sci Rep. 2025 Jan 28;15:3595. doi: 10.1038/s41598-024-83610-1 (PMC11775221; doi:10.1038/s41598-024-83610-1)

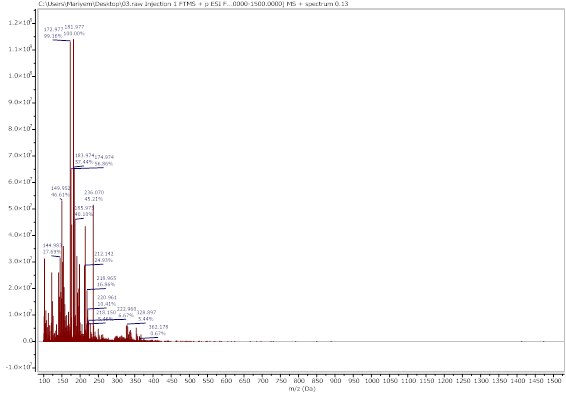


**Figure S1.** Mass spectrum of RhB degradation.

Supplement: Supplementary file 1 — Supplementary Material 1 [file 41598_2024_83610_MOESM1_ESM.docx]
